# Effects of hip stability training versus standard care on Q-angle and pain in marathon runners with patellofemoral pain syndrome: A retrospective cohort study

**DOI:** 10.1371/journal.pone.0357057

**Published:** 2026-09-17

**Authors:** Donghuan Bai, Xiaodong Cao, Shuolei Feng

**Affiliations:** 1 Department of Sports Training, Huaibei Normal University, Huaibei, Anhui, China; 2 Department of Sports, Zhengzhou Technical College, Zhengzhou, Henan, China; 3 Department of Public Course Teaching (Department of Physical Education), Hubei College of the Arts, Wuhan, Hubei, China; 4 Lincoln University College (LUC), Petaling Jaya, Selangor Darul Ehsan, Malaysia; Shahrood University of Technology, IRAN, ISLAMIC REPUBLIC OF

## Abstract

**Objective:**

Hip stability training may influence the quadriceps angle (Q-angle) and pain, but the clinical relevance of static Q-angle changes remains uncertain. This study compared hip stability training with standard care for Q-angle, pain, and function in marathon runners with Patellofemoral Pain Syndrome (PFPS).

**Methods:**

We identified 487 marathon runners with PFPS, of whom 312 met the eligibility criteria. After 1:1 propensity score matching, 94 runners were retained in each group: hip stability training (Hip-STAB) and standard care (Control). Outcomes were assessed at baseline (W0) and at 4, 8, and 16 weeks (W4, W8, W16). Primary outcomes were standing Q-angle, visual analog scale pain during running (VAS-Run; 0–100 mm), and Anterior Knee Pain Scale (AKPS; 0–100 points).

**Results:**

Both groups improved over 16 weeks. Linear mixed-effects models showed significant time effects for Q-angle, VAS-Run, and AKPS (all p < 0.001), but no significant time-by-group interactions for Q-angle (p = 0.190), VAS-Run (p = 0.302), or AKPS (p = 0.317). Bonferroni-adjusted post-hoc comparisons did not identify significant between-group differences at W16. The association between Q-angle reduction and VAS-Run improvement was significant in the Hip-STAB group (r = 0.35, 95% CI 0.16 to 0.52, p < 0.001) and the Control group (r = 0.44, 95% CI 0.26 to 0.59, p < 0.001).

**Conclusions:**

Hip-STAB and standard care were each associated with improvements in static Q-angle, running pain, and function, but Hip-STAB was not superior to standard care. The correlation between Q-angle and pain changes should be interpreted as an association rather than evidence of a causal biomechanical mechanism.

## 1. Introduction

Patellofemoral pain syndrome (PFPS) is one of the most prevalent overuse injuries among marathon runners, characterized by anterior knee pain exacerbated during running and other knee-loading activities [1]. The condition significantly impacts training consistency and performance, with up to 30% of runners experiencing PFPS during their athletic careers [2].

The Q-angle, formed by the intersection of lines from the anterior superior iliac spine to the center of the patella and from the center of the patella to the tibial tuberosity, has been traditionally considered a key biomechanical factor in PFPS development [3]. Increased Q-angles may alter patellofemoral joint tracking, potentially leading to increased joint stress and subsequent pain [4].

Growing evidence suggests that proximal factors, particularly hip muscle function and stability, may influence lower extremity alignment and patellofemoral joint mechanics [5]. Hip abductor and external rotator weakness has been associated with increased femoral adduction and internal rotation during weight-bearing activities, potentially increasing dynamic Q-angles and patellofemoral joint stress [3,6].

Hip stability training programs targeting these muscle groups have emerged as a potentially effective intervention for PFPS [7,8]. However, the comparative effectiveness of hip stability training versus standard care, and the relationship between changes in Q-angle and subsequent pain reduction, remains controversial, with inconsistent findings across studies [9,10].

Most existing research comprises small prospective studies with varying protocols, limiting generalizability. Additionally, few studies have specifically examined these relationships in marathon runners, who experience unique biomechanical demands due to high training volumes and prolonged running durations.

Therefore, this study aimed to compare systematic hip stability training with standard care for changes in Q-angle, running pain, and function in marathon runners with PFPS, using a retrospective cohort design with propensity score matching to reduce measured baseline imbalance. The prespecified directional hypothesis was that Hip-STAB would be associated with greater reductions in Q-angle and pain than standard care; the observational design was not intended to establish causality.

## 2. Methods

### 2.1. Study design and data source

This retrospective cohort study analyzed electronic medical records (EMR) and a rehabilitation database from a single sports medicine center. It was approved by the Institutional Review Board of Lincoln University College (No.: LUC/DVCECA/26022026/023) and was conducted in accordance with the 1964 Helsinki Declaration and its later amendments or comparable ethical standards. Eligible index visits occurred between January 2020 and February 2024. Following Institutional Review Board approval on February 26, 2026, de-identified data were extracted between February 27, 2026 and March 2, 2026. The analytical dataset was finalized and accessed for statistical analysis on March 2, 2026. The requirement for informed consent was waived because the study used retrospective, de-identified clinical data. Marathon runners diagnosed with PFPS were identified through the EMR system using International Classification of Diseases codes. Eligibility was verified through manual chart review by two independent researchers, with disagreements resolved by a third researcher.

### 2.2. Population and eligibility criteria

Eligible participants were marathon runners (defined as individuals who had completed at least one marathon or were training for a marathon with weekly mileage ≥30 miles) diagnosed with PFPS by a sports medicine physician based on established clinical criteria: (1) anterior knee pain aggravated by at least two of the following activities: prolonged sitting, stair climbing, squatting, running, kneeling, or jumping; (2) pain on compression of the patella or palpation of the posterior surface of the patella; (3) absence of other sources of anterior knee pain such as patellar tendinopathy, Osgood-Schlatter disease, or significant internal derangement. Exclusion criteria included: (1) history of knee surgery; (2) patellar dislocation or subluxation; (3) significant internal derangement of the knee joint; (4) osteoarthritis of the patellofemoral or tibiofemoral joint; (5) concurrent lower extremity injury affecting running mechanics; (6) systemic inflammatory disease; (7) incomplete follow-up data; (8) participation in formal physical therapy for PFPS within 6 months prior to baseline assessment. All participants had undergone a comprehensive clinical assessment, including Q-angle measurement, pain evaluation using a visual analog scale during running (VAS-Run), and functional assessment using the Anterior Knee Pain Scale (AKPS) at baseline and follow-up visits. These assessments were performed by trained physical therapists following standardized protocols as part of routine clinical care.

### 2.3. Exposure definition

The exposure of interest was participation in a systematic hip stability training program (Hip-STAB). Based on rehabilitation records, participants were categorized into the Hip-STAB group if they completed at least 4 weeks of a structured hip stability training program including: (1) progressive hip abductor strengthening (side-lying hip abduction, clamshells, monster walks with resistance bands); (2) hip external rotator strengthening; (3) hip extensor strengthening; and (4) functional single-leg stability exercises. The program was prescribed by physical therapists with standardized protocols and performed at least three times weekly.

The Control group received standard care focused on activity modification and quadriceps strengthening without specific emphasis on the hip musculature. The exercise program primarily comprised straight-leg raises and wall squats, with knee flexion during wall squats limited to 0–45 degrees. Participants performed three sets of 10–15 repetitions of each exercise, or three 30–45-second holds for wall squats, three times weekly; each session lasted approximately 20 minutes. Intensity was targeted at a rating of perceived exertion of 4–6 on a 0–10 scale. When three sets of 15 repetitions could be completed without symptom aggravation, resistance was progressed with a 1–2 kg ankle weight or a longer wall-squat hold. Running was permitted when pain remained at or below 3 on a 0–10 pain rating scale and returned to baseline within 24 hours; otherwise, weekly mileage was reduced by approximately 20%−30% and speed work was temporarily suspended. The minimum documented treatment duration was 4 weeks, and continuation through the 16-week follow-up was advised. Adherence was monitored through exercise logs and therapist documentation, and participants completing less than 75% of prescribed sessions were not eligible for the analytical cohort.

### 2.4. Follow-up and outcome measures

**Fig 1:**
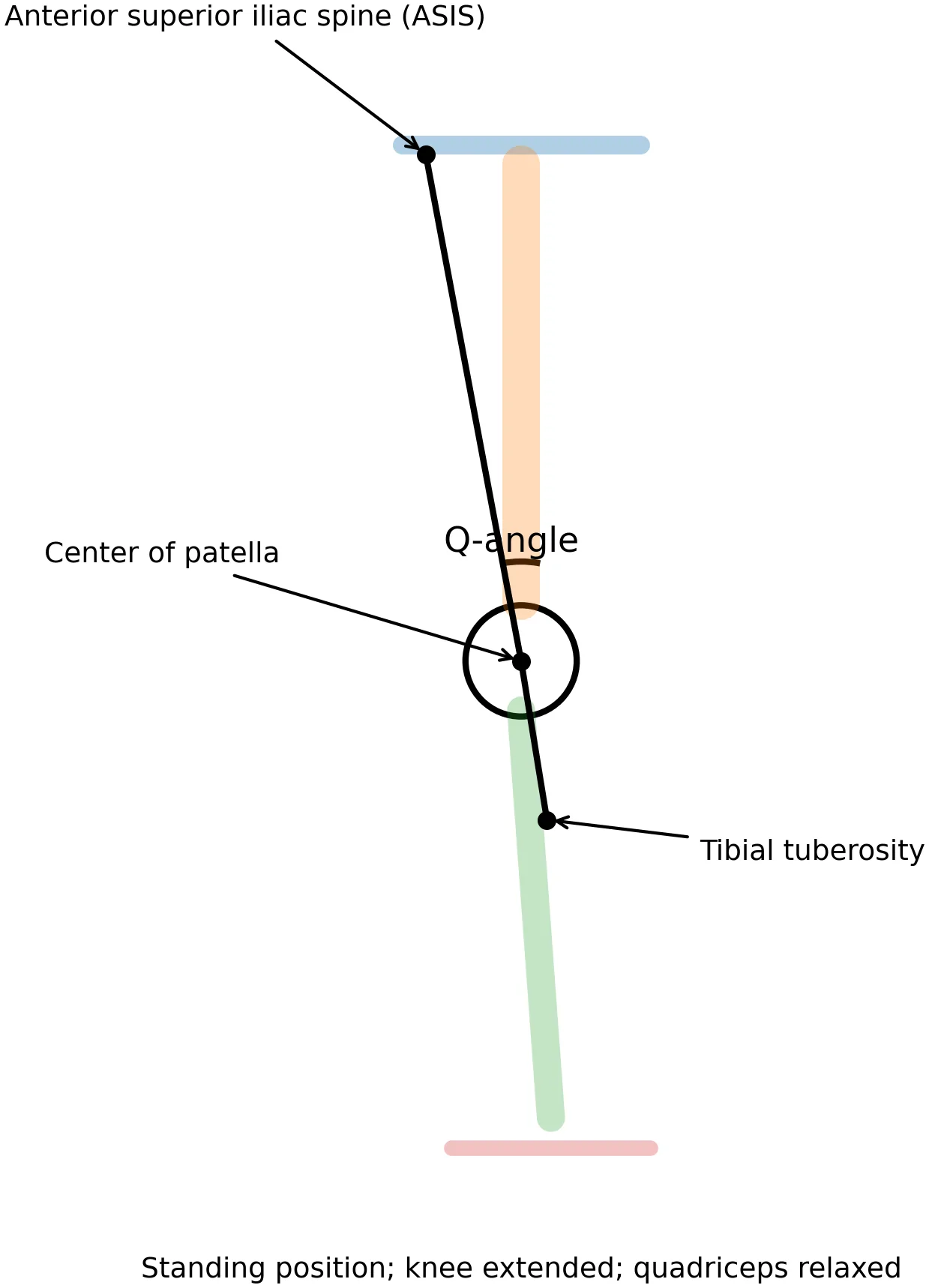
Schematic representation of standing Q-angle measurement. The angle is formed by lines connecting the anterior superior iliac spine, patellar center, and tibial tuberosity while the knee is extended and the quadriceps is relaxed.

Outcomes were assessed at W0, W4, W8, and W16 during clinical follow-up. Q-angle was measured in a static standing position with the knee extended and the quadriceps relaxed, using a digital goniometer. This standardized standing approach has been used in running populations [11], although static clinical Q-angle has recognized limitations relative to dynamic assessment [12]. Two readings were obtained and averaged. The angle was defined by a line from the anterior superior iliac spine to the patellar center and a second line from the patellar center to the tibial tuberosity (Fig 1). Dynamic Q-angle and three-dimensional running biomechanics were not routinely recorded and therefore could not be evaluated. Running pain was assessed on a 0–100 mm VAS-Run, where 0 indicated no pain and 100 the worst pain imaginable. A reduction of at least 20 mm was treated as an exploratory clinically important change based on established PFPS outcome-measure research [13], while recognizing that this threshold has not been validated specifically in marathon runners. Function was measured with the 0–100 AKPS, with higher values indicating better function. Assessor blinding was not documented in the retrospective records and therefore could not be confirmed.

### 2.5. Covariates

Potential confounders collected from medical records were age, sex, body mass index (BMI), running experience, weekly mileage, previous PFPS episodes, bilateral symptoms, symptom duration, knee valgus during a single-leg squat, foot posture index, and non-steroidal anti-inflammatory drug use.

### 2.6. Statistical analysis

Propensity scores were estimated with logistic regression including all prespecified measured covariates, explicitly including knee valgus during the single-leg squat, and 1:1 nearest-neighbor matching used a caliper of 0.2 standard deviations of the logit of the propensity score. Balance was evaluated with standardized mean differences (SMD), with SMD < 0.1 indicating acceptable balance. Q-angle, VAS-Run, and AKPS were analyzed using linear mixed-effects models with categorical time, group, and time-by-group interaction as fixed effects and participant-specific random intercepts. Five planned between-group contrasts (W0, W4, W8, W16, and W0-W16 change) were Bonferroni-adjusted; adjusted p-values and 99% confidence intervals are reported. Pearson correlations between Q-angle and VAS-Run changes were calculated overall and separately within each treatment group. Multiple linear regression evaluated the association between W0-W16 Q-angle reduction and VAS-Run improvement while adjusting for group, age, sex, BMI, running experience, weekly mileage, previous PFPS, bilateral symptoms, and baseline VAS-Run. The 20-mm VAS-Run threshold was examined with chi-square tests. No a priori sample-size calculation was performed because all eligible records in the study period were analyzed. Statistical significance was set at p < 0.05 for primary model effects. Analyses were conducted in Python version 3.13.5 using statsmodels version 0.14.6 and SciPy version 1.17.0.

## 3. Results

### 3.1. Participant flow

**Fig 2:**
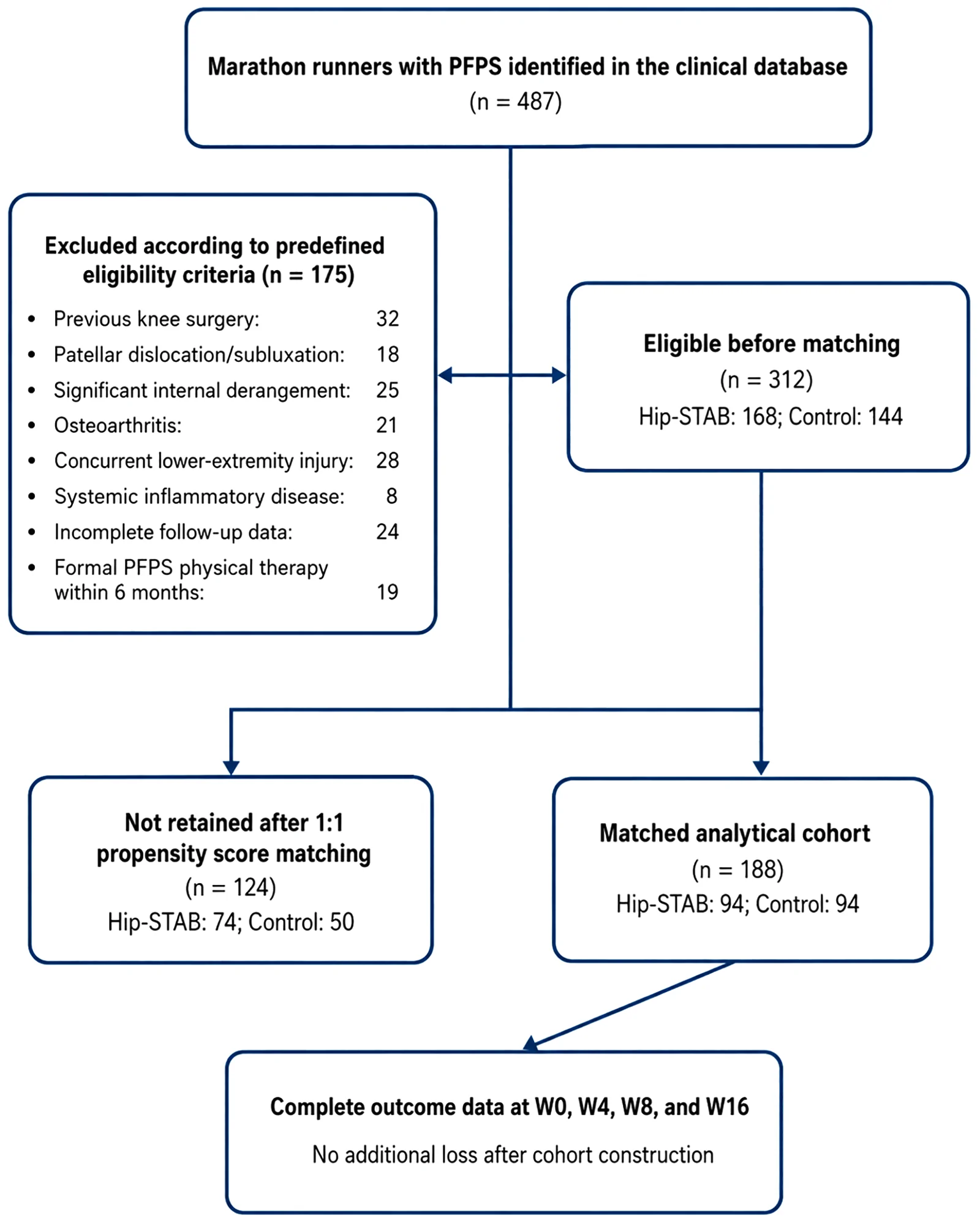
Participant identification, exclusion, propensity score matching, and final analytical cohort.

A total of 487 marathon runners were initially identified. Of these, 175 were excluded based on the predefined eligibility criteria: previous knee surgery (n = 32), patellar dislocation or subluxation (n = 18), significant internal knee derangement (n = 25), patellofemoral or tibiofemoral osteoarthritis (n = 21), concurrent lower-extremity injury affecting running mechanics (n = 28), systemic inflammatory disease (n = 8), incomplete follow-up data (n = 24), and formal physical therapy for PFPS within the preceding 6 months (n = 19). This resulted in 312 eligible participants before matching, including 168 in the Hip-STAB group and 144 in the Control group. Following propensity score matching, 124 participants were not retained because no suitable match was identified (74 Hip-STAB; 50 Control). The final matched cohort contained 188 participants (94 per group). All 188 had complete W0, W4, W8, and W16 outcome data, with no additional loss after cohort construction (Fig 2).

### 3.2. Baseline characteristics

Before matching, Hip-STAB participants were younger, had lower BMI, and reported greater weekly mileage than Control participants, consistent with possible treatment self-selection by younger and fitter runners. After matching, all measured baseline characteristics were balanced with SMDs < 0.1 (Table 1); nevertheless, propensity score matching cannot eliminate imbalance in unmeasured factors such as motivation, treatment preference, or health-seeking behavior.

**Table 1:** Baseline characteristics before and after propensity score matching.

Characteristic	Before Matching			After Matching		
	Hip-STAB (n = 168)	Control (n = 144)	SMD	Hip-STAB (n = 94)	Control (n = 94)	SMD
Age, years	34.2 ± 8.7	38.5 ± 10.3	0.45	36.3 ± 9.1	36.8 ± 9.3	0.05
Sex, female	92 (54.8%)	68 (47.2%)	0.15	48 (51.1%)	50 (53.2%)	0.04
BMI, kg/m²	22.4 ± 2.6	23.8 ± 3.1	0.49	23.1 ± 2.8	23.2 ± 2.9	0.03
Running experience, years	6.8 ± 4.3	5.9 ± 5.1	0.19	6.2 ± 4.5	6.3 ± 4.7	0.02
Weekly mileage, miles	42.7 ± 15.3	36.2 ± 14.1	0.44	38.9 ± 13.8	38.5 ± 14.0	0.03
Previous PFPS episodes	72 (42.9%)	68 (47.2%)	0.09	42 (44.7%)	44 (46.8%)	0.04
Bilateral symptoms	58 (34.5%)	54 (37.5%)	0.06	34 (36.2%)	35 (37.2%)	0.02
Symptom duration, weeks	8.3 ± 6.7	10.2 ± 8.1	0.26	9.1 ± 7.2	9.3 ± 7.5	0.03
Knee valgus in single-leg squat	98 (58.3%)	76 (52.8%)	0.11	52 (55.3%)	54 (57.4%)	0.04
Foot posture index	3.2 ± 4.1	2.9 ± 4.3	0.07	3.0 ± 4.2	3.1 ± 4.1	0.02
NSAID use	64 (38.1%)	58 (40.3%)	0.04	37 (39.4%)	38 (40.4%)	0.02

Data presented as mean ± SD or count (percentage). SMD = standardized mean difference; BMI = body mass index; PFPS = patellofemoral pain syndrome; NSAID = non-steroidal anti-inflammatory drug.

### 3.3. Q-angle changes

**Fig 3:**
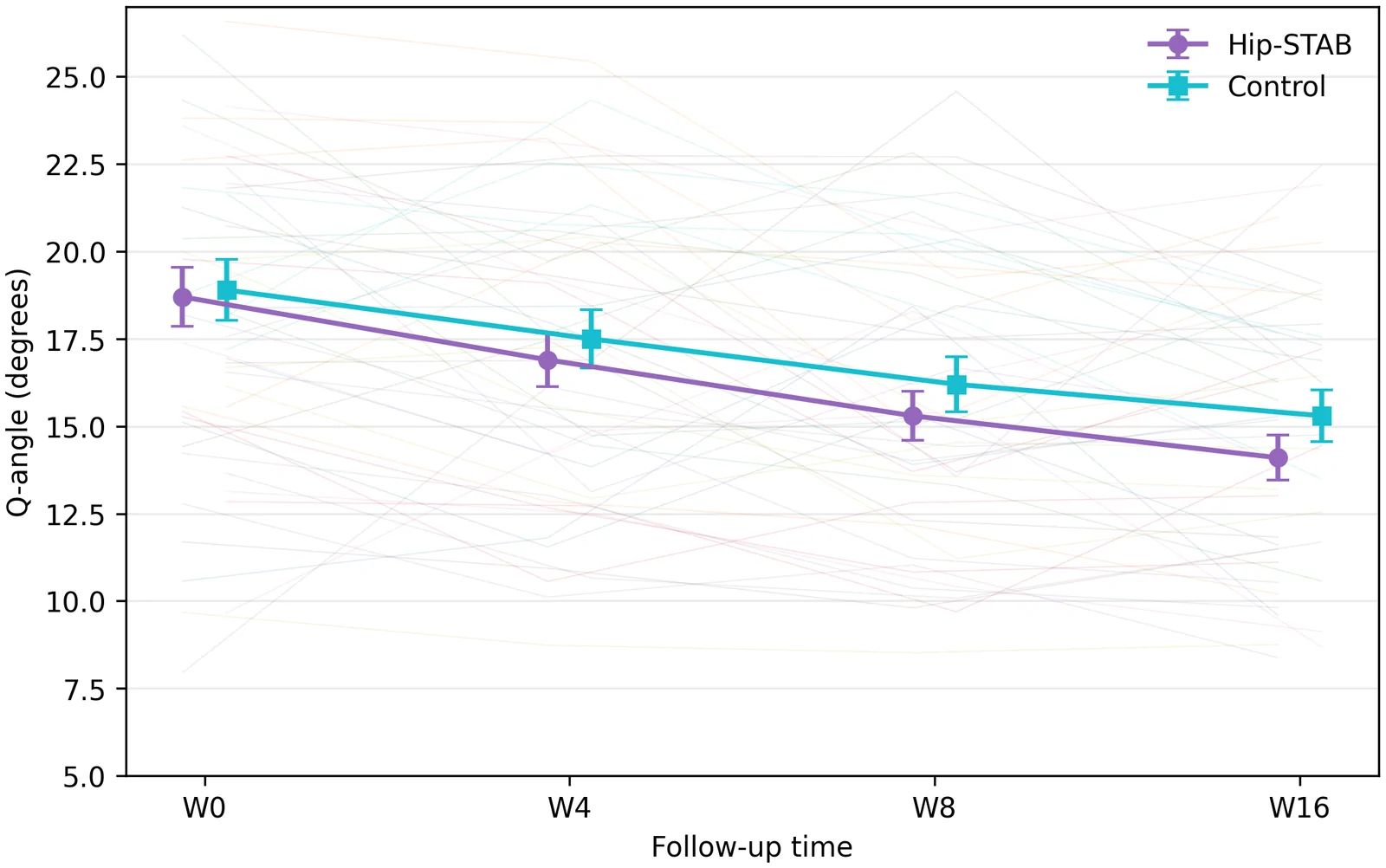
Q-angle measurements at W0, W4, W8, and W16. Lines show group means with 95% confidence intervals; faint trajectories show individual participants. Q-angle was measured in standing with the knee extended and quadriceps relaxed.

As shown in Fig 3 and Table 2, both groups demonstrated progressive reductions in Q-angle. The mixed-effects model showed a significant time effect (p < 0.001), a non-significant group coefficient at W0 (p = 0.721), and no significant time-by-group interaction (p = 0.190). The W16 between-group contrast was not significant after Bonferroni adjustment (adjusted p = 0.159).

**Table 2:** Changes in Q-angle (degrees) from baseline.

Time Point	Hip-STAB (n = 94)	Control (n = 94)	Mean Difference (99% CI)	Bonferroni-adjusted p-value	Effect Size (Cohen’s d)
W0 (Baseline)	18.7 ± 4.2	18.9 ± 4.3	−0.2 (−1.6 to 1.2)	1.000	0.05
W4	16.9 ± 3.8	17.5 ± 4.1	−0.6 (−2.0 to 0.8)	1.000	0.15
W8	15.3 ± 3.5	16.2 ± 3.9	−0.9 (−2.3 to 0.5)	0.537	0.24
W16	14.1 ± 3.2	15.3 ± 3.7	−1.2 (−2.6 to 0.2)	0.159	0.34
Change W0-W16	−4.6 ± 2.7	−3.6 ± 2.9	−1.0 (−2.2 to 0.2)	0.185	0.36

Data are mean ± SD. Between-group contrasts report Bonferroni-adjusted p-values and 99% confidence intervals for five planned comparisons.

### 3.4. Pain during running (VAS-Run)

**Fig 4:**
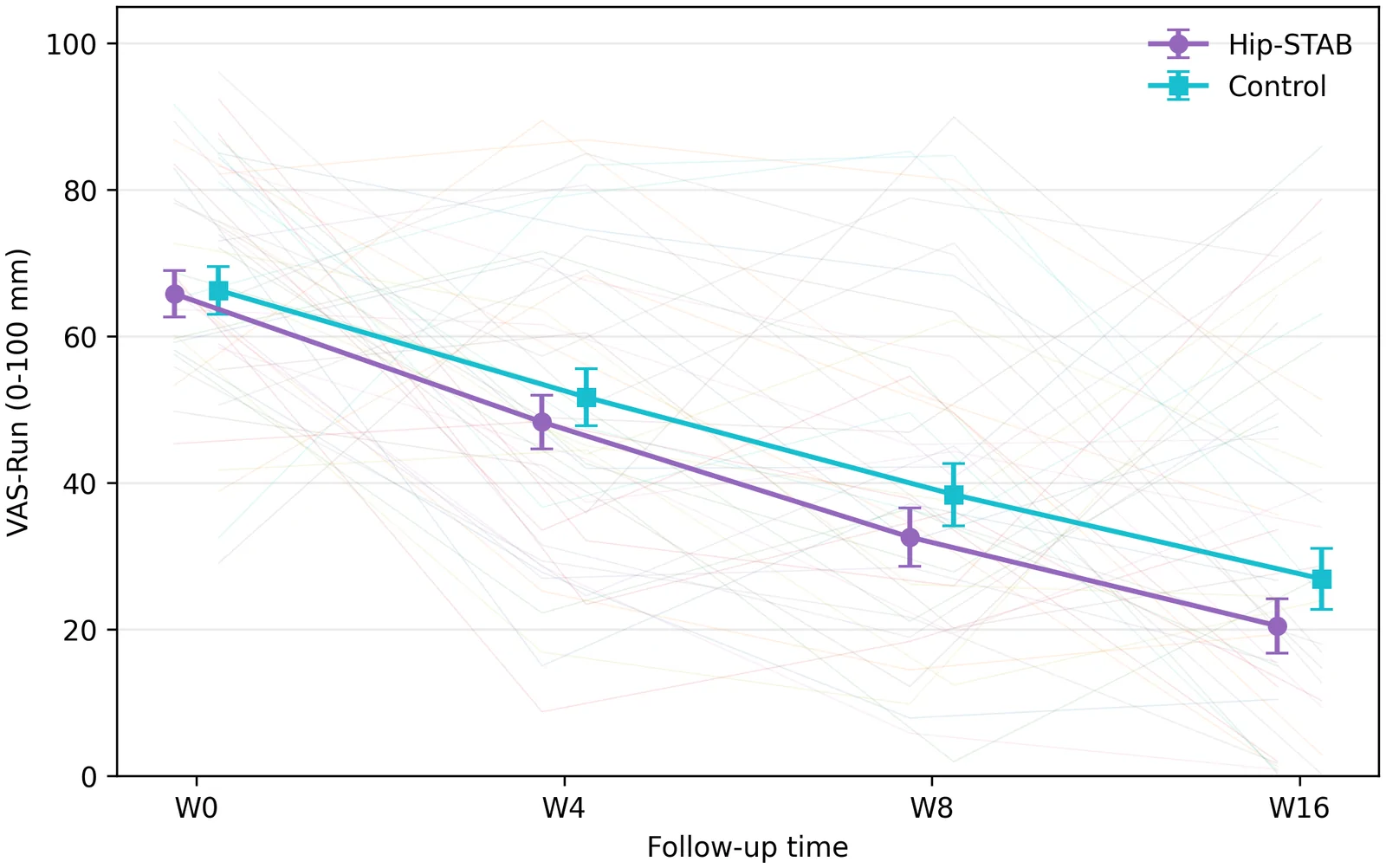
VAS-Run scores at W0, W4, W8, and W16. Lines show group means with 95% confidence intervals; faint trajectories show individual participants. VAS-Run ranges from 0 to 100 mm, with lower values indicating less pain.

Both groups showed substantial reductions in running pain over time (Fig 4, Table 3). The mixed-effects model showed a significant time effect (p < 0.001), a non-significant group coefficient at W0 (p = 0.854), and no significant time-by-group interaction (p = 0.302). The numerically lower W16 VAS-Run value in Hip-STAB did not remain significant after Bonferroni adjustment (adjusted p = 0.094) and cannot be interpreted as treatment superiority.

**Table 3:** Changes in VAS-Run scores (0-100 mm) from baseline.

Time Point	Hip-STAB (n = 94)	Control (n = 94)	Mean Difference (99% CI)	Bonferroni-adjusted p-value	MCID Achievement
W0 (Baseline)	65.8 ± 15.7	66.3 ± 16.1	−0.5 (−7.5 to 6.5)	1.000	–
W4	48.3 ± 18.2	51.7 ± 19.4	−3.4 (−10.4 to 3.6)	1.000	43 (45.7%) vs. 38 (40.4%), p = 0.461
W8	32.6 ± 19.8	38.4 ± 21.2	−5.8 (−12.8 to 1.2)	0.166	67 (71.3%) vs. 58 (61.7%), p = 0.164
W16	20.5 ± 18.4	26.9 ± 20.7	−6.4 (−13.4 to 0.6)	0.094	79 (84.0%) vs. 72 (76.6%), p = 0.199
Change W0-W16	−45.3 ± 21.6	−39.4 ± 22.9	−5.9 (−14.8 to 3.0)	0.432	–

Data are mean ± SD. Between-group contrasts report Bonferroni-adjusted p-values and 99% confidence intervals for five planned comparisons. MCID = minimal clinically important difference, defined as a reduction of at least 20 mm.

### 3.5. Functional status (AKPS)

**Fig 5:**
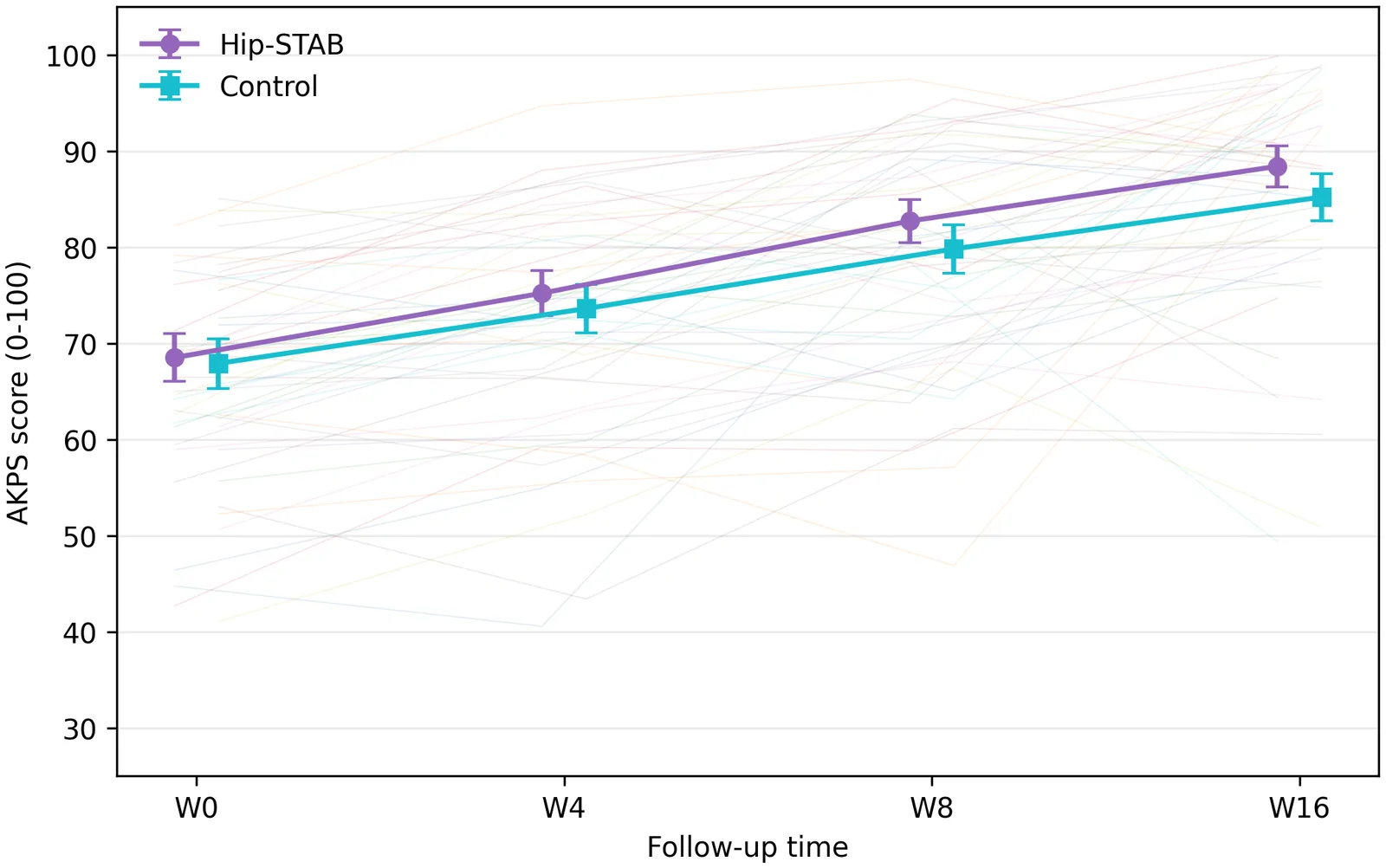
AKPS scores at W0, W4, W8, and W16. Lines show group means with 95% confidence intervals; faint trajectories show individual participants. Higher AKPS scores indicate better function.

Both groups demonstrated improved AKPS scores over time (Fig 5, Table 4). The mixed-effects model showed a significant time effect (p < 0.001), a non-significant group coefficient at W0 (p = 0.730), and no significant time-by-group interaction (p = 0.317). The W16 between-group contrast was not significant after Bonferroni adjustment (adjusted p = 0.327).

**Table 4:** Changes in AKPS scores (0-100) from baseline.

Time Point	Hip-STAB (n = 94)	Control (n = 94)	Mean Difference (99% CI)	Bonferroni-adjusted p-value	Effect Size (Cohen’s d)
W0 (Baseline)	68.5 ± 12.3	67.9 ± 12.8	0.6 (−3.9 to 5.1)	1.000	0.05
W4	75.2 ± 11.7	73.6 ± 12.4	1.6 (−2.9 to 6.1)	1.000	0.13
W8	82.7 ± 11.2	79.8 ± 12.6	2.9 (−1.6 to 7.4)	0.475	0.24
W16	88.4 ± 10.5	85.2 ± 12.1	3.2 (−1.3 to 7.7)	0.327	0.28
Change W0-W16	19.9 ± 11.4	17.3 ± 12.8	2.6 (−1.4 to 6.6)	0.488	0.22

Data are mean ± SD. Between-group contrasts report Bonferroni-adjusted p-values and 99% confidence intervals for five planned comparisons. AKPS = Anterior Knee Pain Scale.

### 3.6. Correlation and regression analysis

Exploratory analyses showed that W0-W16 Q-angle reduction was moderately associated with VAS-Run improvement overall (r = 0.41, 95% CI 0.28 to 0.52, p < 0.001). The association remained significant when analyzed separately in Hip-STAB (r = 0.35, 95% CI 0.16 to 0.52, p < 0.001) and Control (r = 0.44, 95% CI 0.26 to 0.59, p < 0.001) participants (Table 5).

**Table 5:** Correlation between changes in Q-angle and changes in pain (VAS-Run).

Time interval	Overall r (95% CI), p	Hip-STAB r (95% CI), p	Control r (95% CI), p
W0-W4	0.22 (0.08 to 0.35), p = 0.002	0.15 (−0.05 to 0.34), p = 0.145	0.26 (0.06 to 0.44), p = 0.010
W0-W8	0.34 (0.21 to 0.46), p < 0.001	0.24 (0.04 to 0.42), p = 0.018	0.39 (0.21 to 0.55), p < 0.001
W0-W16	0.41 (0.28 to 0.52), p < 0.001	0.35 (0.16 to 0.52), p < 0.001	0.44 (0.26 to 0.59), p < 0.001

CI = confidence interval. Positive coefficients indicate that greater Q-angle reduction was associated with greater VAS-Run improvement.

In the adjusted linear regression, greater Q-angle reduction remained associated with greater VAS-Run improvement (beta = 4.64 mm per degree, 95% CI 3.68 to 5.60, p < 0.001), whereas treatment group was not independently associated with pain improvement (beta = 1.19 mm, 95% CI −3.49 to 5.87, p = 0.617) (Table 6). These observational associations do not establish the direction of effect.

**Table 6:** Multiple linear regression model for change in VAS-Run from baseline to Week 16.

Variable	β Coefficient (95% CI)	p-value
Change in Q-angle (degrees)	4.64 (3.68 to 5.60)	<0.001
Group (Hip-STAB vs. Control)	1.19 (−3.49 to 5.87)	0.617
Age (years)	0.25 (−0.03 to 0.53)	0.076
Sex (female vs. male)	−4.98 (−10.19 to 0.24)	0.061
BMI (kg/m²)	−0.86 (−1.82 to 0.11)	0.081
Running experience (years)	−0.39 (−1.03 to 0.25)	0.227
Weekly mileage (miles)	0.11 (−0.08 to 0.29)	0.266
Previous PFPS episodes (yes vs. no)	−4.58 (−9.71 to 0.55)	0.080
Bilateral symptoms (yes vs. no)	−5.01 (−10.44 to 0.43)	0.071
Baseline VAS-Run score	0.45 (0.28 to 0.62)	<0.001

CI = confidence interval; BMI = body mass index; PFPS = patellofemoral pain syndrome; VAS-Run = visual analog scale for pain during running. Adjusted R² = 0.501.

### 3.7. Adverse events

No serious adverse events were reported in either group. Minor adverse events included temporary increases in knee pain (4 participants in the Hip-STAB group and 5 in the Control group) and muscle soreness (7 participants in the Hip-STAB group and 3 in the Control group). These symptoms were transient and resolved with temporary modification of the exercise program.

## 4. Discussion

This retrospective cohort study compared Hip-STAB with standard care for static Q-angle, running pain, and function in marathon runners with PFPS. Both groups improved over 16 weeks, but the primary time-by-group interactions were not significant and Hip-STAB was not superior to standard care. Q-angle reduction and pain improvement were moderately correlated in both treatment groups; this association is hypothesis-generating and does not demonstrate that changing static Q-angle caused pain relief.

The absence of a differential treatment effect is consistent with evidence that hip-focused and quadriceps-focused exercise programs can yield comparable improvements for some individuals with PFPS [14], although meta-analytic evidence also supports combined hip and knee strengthening over knee exercise alone in broader PFPS populations [15]. The present findings therefore support exercise therapy while not establishing one program as uniformly superior.

Both study groups received active exercise-based care, which may explain the strong time effects. Contemporary guidance identifies exercise therapy as a core component of PFPS management and emphasizes individualized combinations of education, hip and knee exercise, and other interventions rather than a single universally superior protocol [16–18].

PFPS is multifactorial, and treatment response may depend on symptom duration, movement patterns, psychosocial factors, training exposure, and patient expectations [19–21]. The marked pre-matching differences in age, BMI, and weekly mileage suggest that younger and fitter runners may have preferentially selected Hip-STAB. Matching balanced the measured variables, but motivation and treatment preference were unavailable and could still have influenced adherence and outcomes.

The correlations between Q-angle and pain changes should not be interpreted as evidence of a biomechanical causal pathway. Reverse causality is plausible: pain reduction may permit altered movement, greater loading tolerance, or different lower-limb positioning, which could subsequently change the static Q-angle measurement. Shared responses to exercise, neuromuscular control, or other unmeasured processes may also explain the association.

Static Q-angle is an incomplete surrogate for the dynamic mechanics of running. Dynamic knee valgus can discriminate individuals with PFPS more effectively than clinical static Q-angle in some settings [12], and clinical Q-angle does not reliably represent in-vivo quadriceps force direction or patellofemoral kinematics [22]. Consequently, the present data should be interpreted as changes in a static clinical alignment measure rather than direct evidence of altered running biomechanics.

Clinically, both Hip-STAB and standard care were associated with meaningful improvement over time. The results support flexibility in selecting an exercise program according to the runner’s impairments, preferences, access, and tolerance, but they do not justify recommending Hip-STAB as superior. The higher frequency of transient muscle soreness in Hip-STAB (7 versus 3 participants) also indicates that progression and symptom monitoring are important when prescribing additional hip-loading exercises.

Strengths include the matched cohort, complete four-time-point outcome data, and a 16-week follow-up in a specific athletic population. Propensity score matching reduced imbalance in measured baseline characteristics but does not replicate randomization and cannot control unmeasured confounding.

### 4.1. Limitations

The retrospective design limited control over intervention delivery, assessor blinding, and completeness of clinical documentation. Assessor blinding was not recorded, introducing possible measurement bias. Pre-matching differences suggest treatment self-selection, and residual confounding by motivation, preference, or adherence remains possible despite propensity score matching. Static standing Q-angle does not capture dynamic knee valgus, multiplanar lower-limb motion, or patellofemoral loading during running, substantially reducing the ecological validity and biomechanical interpretability of the findings for marathon runners. The 20-mm VAS threshold was derived from general PFPS research rather than validated specifically for marathon runners. Exercise implementation likely varied across patients and therapists, and the single-center design limits generalizability. Transient muscle soreness occurred more often in Hip-STAB, although no serious adverse events were observed.

### 4.2. Future directions

Future prospective randomized trials should combine static clinical measures with three-dimensional running analysis, dynamic knee valgus, hip strength, return-to-run outcomes, recurrence, and longer follow-up. Broader rehabilitation models should also consider psychological adjuncts such as mindfulness [23], distal loading factors including plantar pressure and foot muscle function [24], training and psychological predictors of running injury [25], scalable multicomponent online programs [26], and dynamic postural control [27]. Such studies could identify clinically relevant subgroups and clarify whether neuromuscular or dynamic movement changes mediate pain improvement.

## 5. Conclusion

In marathon runners with PFPS, Hip-STAB and standard care were each associated with improved static Q-angle, running pain, and function over 16 weeks, with no significant evidence that Hip-STAB was superior. The observed correlations between Q-angle and pain changes were present in both groups but do not establish causality or a specific biomechanical mechanism. Exercise selection should therefore be individualized, and conclusions about running mechanics require prospective studies with dynamic biomechanical assessment.
